# The immunomodulatory role of tumor-initiating cells in digestive system tumors: from mechanisms to therapy

**DOI:** 10.3389/fimmu.2025.1621464

**Published:** 2025-07-24

**Authors:** Zun-yue Zhang, Xin-feng Zhang, Cong-hui Xu, Kun-hua Wang, Fang Huang

**Affiliations:** ^1^ School of Medicine, Yunnan University, Kunming, China; ^2^ Yunnan Technological Innovation Centre of Drug Addiction Medicine, Yunnan University, Kunming, China; ^3^ Department of Gastrointestinal and Hernia Surgery, The First Affiliated Hospital of Kunming Medical University, Kunming, China

**Keywords:** tumor-initiating cells (TICs), digestive system tumors, immunotherapy, tumor microenvironment, immune checkpoints, combination therapy, differentiation therapy

## Abstract

Targeting tumor-initiating cells (TICs) in digestive system tumors is a feasible strategy to boost the effectiveness of cancer immunotherapy. Because of their stem cell-like properties, TICs can cause tumor heterogeneity, recurrence, and resistance to conventional medicines, which can seriously impair treatment outcomes. This review discusses the unique features of TICs inside various digestive system tumors, such as colorectal, pancreatic, liver, and gastric cancers. We look at the mechanisms that TICs evade immune recognition, including altered tumor microenvironment, decreased immunogenicity, and immune checkpoint molecule expression. Furthermore, we highlight potential strategies for TICs, such as differentiation therapies, inhibiting certain signaling pathways, and enhancing immune recognition through advanced immunotherapeutic approaches. The analysis also examines the potential for combination therapy, which include adoptive cell therapies, TIC-targeted strategies, and immune checkpoint inhibitors. Lastly, we address the challenges presented by TIC heterogeneity and immune escape mechanisms, emphasizing the need for more clinical research to back up these innovative tactics. All things considered, TIC targeting is a significant method to improve immunotherapy’s efficacy in treating digestive system cancers, which will ultimately help patients.

## Introduction

Along with the great majority of non-tumorigenic cells, some tumors have a little number of cells that have the ability to self-renew and start new tumors. Tumor-initiating cells (TICs), also called cancer stem cells (CSCs) or CSC-like cells, are a group of cells that can produce diverse cell populations that closely mimic the original tumor’s makeup (1). TICs, a subset of cells within malignancies, may arise from alterations in progenitor or normal stem cells or from genetic defects. Because of their similar ability to differentiate into diverse lineages, TICs were formerly thought to be produced from normal stem cells. Transduction of the MLL-AF9 fusion protein into myeloid progenitor cells can generate leukemia *in vivo*, indicating that progenitor cells can develop into leukemic stem cells (2). Subsequent research has demonstrated that TICs can be created by genetically altering tumor endothelial cells and astrocytes to dedifferentiate (3, 4). This suggests that TICs may originate from a variety of sources, which could be a major factor in their differentiation from healthy stem cells. TICs differ from normal stem cells in their phenotypes and capabilities. TICs and normal stem cells are two distinct cell types with different characteristics and roles. Despite their similarities, they nevertheless differ significantly, particularly in terms of behavior, regulation, and their effects on health and illness (5). Normal stem cells help replace damaged or dying cells and maintain tissue homeostasis. TICs are believed to be responsible for tumor genesis, growth, and recurrence due to their stem-like properties, which leads to intratumor heterogeneity of cancer cells (6). While TICs can self-renew and manufacture more TICs and differentiated cancer cells, normal stem cells can divide and produce identical stem cells and differentiated cells. Normal stem cell division is tightly regulated by internal mechanisms to prevent excessive proliferation and maintain tissue integrity in response to specific stimuli from the milieu (the stem cell niche). However, if left untreated, TICs multiply, leading to the growth and advancement of malignancies (7). Normal stem cells frequently maintain genomic stability, have low rates of genetic mutation, and can efficiently repair DNA damage, whereas TICs directly contribute to intratumor heterogeneity (8, 9).

Whether TICs represent a distinct group of cancer cells or a functional state of some cancer cells is still up for dispute. TICs are now widely recognized as being crucial to therapeutic progress due to their ability to self-renew, resist chemotherapy, and respond to immune checkpoint inhibitors (10–12). The TIC concept has led to a reexamination of therapeutic alternatives for cancer cure. The issues of cancer recurrence and metastatic spread still require attention, even though many malignancies have been adequately remitted by a number of popular anti-cancer medicines. Most conventional medications are cytotoxic and highly non-selective because their aim is to destroy all rapidly proliferating cells. Sometimes, this approach can lead to a difference between a good clinical response (a significant reduction in tumor size) and an inadequate survival response. This could be due to TIC-driven recurrence after a significant number of cancer cells are killed without totally removing the TICs (13, 14). Digestive system tumors offer a fantastic chance to increase the efficacy of cancer immunotherapy. TIC is a cell population of extremely drug-resistant, asymmetrically dividing, tumor-initiating cells that arises after early success of tumor radiotherapy or chemotherapy. It has a strong correlation with tumor heterogeneity and is essential for clinical phenomena such as treatment resistance, tumor metastasis, tumor dormancy, and recurrence (12, 15). Digestive system tumors, such as those of the stomach, liver, pancreas, and colon, present a challenging issue since they usually exhibit resistance to standard therapies. Resistance (16–18), recurrence, and metastasis (3, 19) are believed to be influenced by the unique features of TICs, including their ability to self-renew, differentiate, and evade the immune system. Therefore, if the characteristics of TICs are well defined, the reasons for their variability and immune evasion mechanisms are investigated, and potential indicators are identified, targeting TICs may help cure digestive system cancers.

## TICs in digestive system tumors

There is a subgroup of tumor cells called TICs that share traits with stem cells. Because they may self-renew and create the heterogeneity of tumor cells, they are important in tumor spread and recurrence. The TIC markers and their functions in gastrointestinal malignancies, including colorectal, stomach, liver, and pancreatic cancers, are listed in **Table 1**. TICs are often resistant to chemotherapy and radiation due to their quiescent nature, enhanced DNA repair processes, and ability to evade the immune system. They are therefore a crucial target for the efficacy of long-term treatment.

**Table 1:** Stem cell biomarkers and functions identified in gastrointestinal tumors.

Cancers	Maker	Validation method	Function
Gastric cancer	CD90 (20)	*in vitro*	CD90: belongs to the immunoglobulin superfamily and the Thy-1 cell-surface antigen (adhesion molecule) family, which is involved in several signal pathways
	CD71 (21)	clinical	CD71: correlation with GC invasion
	CD44 (22)	*in vitro* *in vivo* clinical	CD44: A glycoprotein involves in cell migration and self-renewal
	CXCR4 (23)	*in vitro*	CXCR4:CXCR4+ cells can form spheroid colonies, and they have high metastatic ability and chemotherapy resistance *in vitro*. Moreover, CXCR4+ cells have tumorigenicity and TICs generation capacity in immune-deficient mice *in vivo*.
	CD133 (24)	clinical	CD133: A transmembrane glycoprotein that maintains lipid composition in cell membranes
	LGR5 (25)	clinical	LGR5: lymph node metastasis associated with GC
	MIST1 (26)	*in vivo*	MIST1: related to the proliferation and regeneration of gastric cells
	ALDH1 (27)	clinical	ALDH1: belongs to the aldehyde dehydrogenase family involves in cell migration and self-renewal
	AQP5 (28)	clinical	AQP5: synergizes with LGR5
	CD24 (29)	*in vitro*	CD24: correlation with the advanced stages, invasiveness, and lymph node metastasis of GC.
	CD49f (30)	clinical	CD49f:associated with gastric carcinogenesis and drug resistance
	CD54 (31)	clinical	CD54: associated with chemotherapy resistance
Colorectal cancer	CD49f (32), CD133 (33) CD44 (34), LGR5 (35)	*in vitro* *in vivo* clinical	
	CD200 (36)	*in vitro*	CD200: Interactions between CD200 and its receptor CD200R act as an immune tolerance signal, which reduces myeloid cell activity and change their migration ability
	Ep CAM (35)	*in vitro* clinical	Ep CAM: functions in cell signaling, differentiation, proliferation and migration.
	LGR4 (35)	*in vitro* clinical	LGR4: expression levels of LGR4 was correlated to poor prognosis in CRC patients.
	ALDH (37)	*in vitro* *in vivo* clinical	
	CD166 (37)	*in vitro* *in vivo* clinical	CD166: protect cells against apoptosis and autophagy.
	CD206 (38)	clinical	CD206: involved in endogenous molecule clearance, antigen presentation, and modulation of cellular activity.
Pancreatic cancer	CD133 (39), CD44	*in vitro*	
	CD24 (40)	clinical	CD24: cell surface protein involved in cell adhesion.
	DCLK1 (41)	*in vivo*	DCLK1: induces deregulation of VEGF-inhibitors and leads to neovascularization.
	CXCR4 (42),Ep CAM (43)	*in vivo* clinical	
	Oct4 (44)	*in vitro*	Oct4: the main factor in pluripotency, participating in cell differentiation, reprogramming and renewal.
	ABCB1 (45)	*in vitro*	ABCB1: involved in the resistance of pancreatic cells.
Hepatocellular carcinoma	CD90 (46)	clinical	CD90: involved in cell-cell and cell-matrix interactions.
	EpCAM (47), CD24 (48)	clinical clinical	
	CD44 (49), CD133 (49)	*in vitro*	
	CD13 (50)	*in vivo*	CD13: associated with chemotherapy resistance

## Mechanisms of immune evasion by TICs

### Immunosuppressive microenvironment

**Figure 1:**
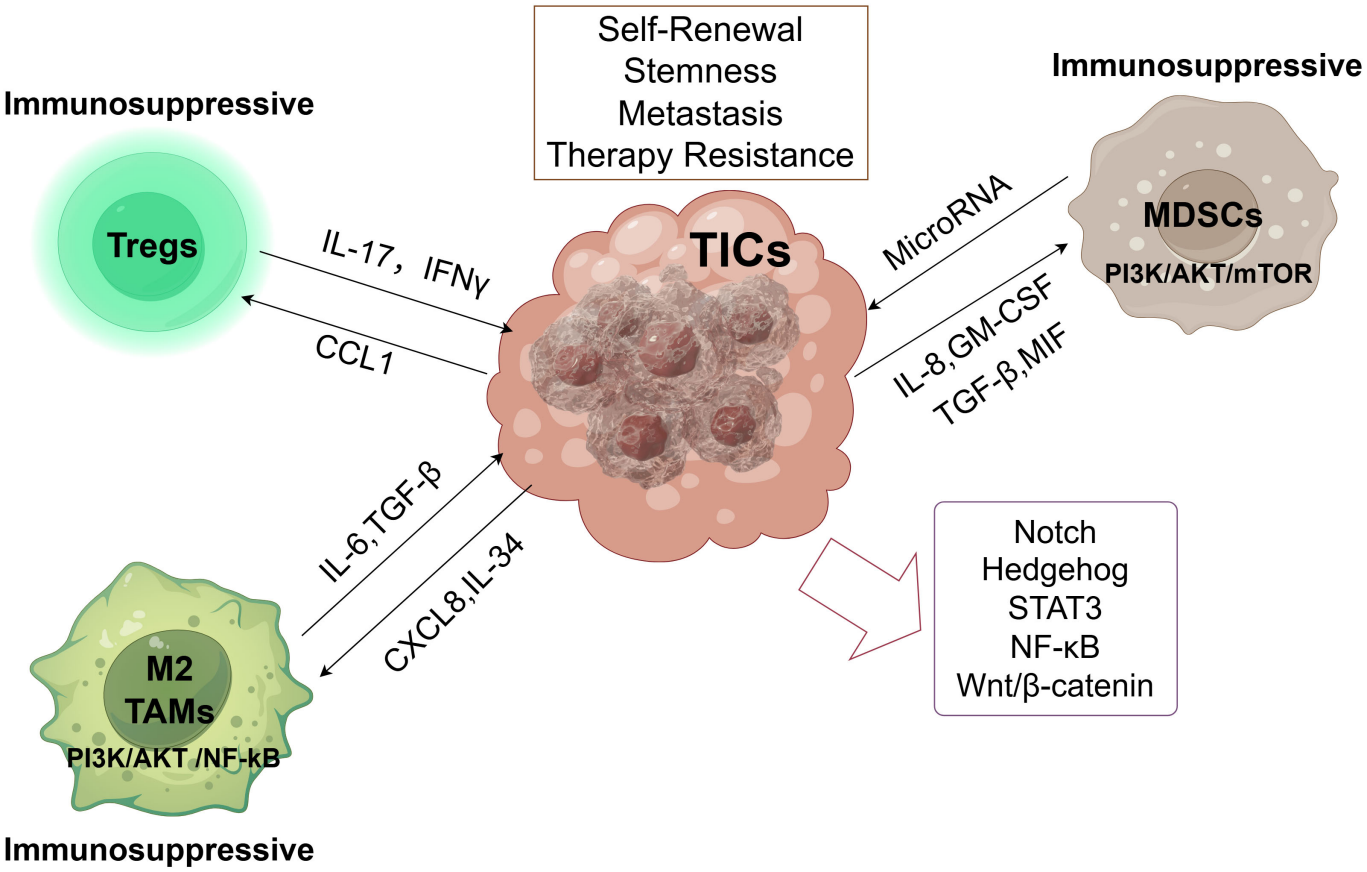
Tumor-initiating cells (TICs) orchestrate an immunosuppressive microenvironment. By releasing several soluble factors, TICs create an immunosuppressive environment that attracts MDSCs, TAMs, and Tregs to the tumor microenvironment. By encouraging tumor angiogenesis, chemoresistance, and tumor spreading, these immunosuppressive cells create an immunosuppressive environment. More significantly, they preserve the stemness and functionality of TICs. These immunosuppressive cells promote TIC self-renewal, stemness, metastasis, and therapy resistance, primarily through PI3K/AKT/mTOR signaling (MDSCs) and PI3K/AKT/NF-κB signaling (M2 TAMs). Key signaling pathways (Notch, Hedgehog, STAT3, NF-κB, Wnt/β-catenin) active within TICs regulate their core properties.

The tumor microenvironment (TME) is the primary site of conflict between tumor cells and the host immune system. Numerous studies have examined the relationship between immune modulation and cancer, specifically cancer dormancy (51). Non-cellular and cellular elements, including immune cells, cancer-associated fibroblasts, and other stromal cells, as well as secretomes and exosomes generated from these cells, comprise the TME. Together, these elements provide an environment that suppresses the immune system and promotes tumor growth. The TME is essentially a cellular environment based on tumors or TICs that significantly encourages the unchecked proliferation of tumor cells or TICs, which in turn influences the host system’s capacity to develop cancer (52). Immunotherapy stimulates the immune system to attack cancer cells in the TME. It aims to boost the activity of natural killer (NK) cells and cytotoxic CD8+ T lymphocytes (CTLs), as opposed to immunosuppressive cells such as mesenchymal-derived suppressor cells (MDSCs), regulatory T cells (Tregs), tumor-associated macrophages (TAMs), and cancer-associated fibroblasts (CAFs) (53). The failure of cancer immunotherapy is often due to the fact that TICs can overcome anti-tumor immunity (54). TICs can evade tumor immunity by changing the TME, and these environmental changes can result in TIC phenotypic changes that facilitate tumor immune evasion (55, 56). For instance, regulatory myeloid cells, such as Tregs and MDSCs, are drawn to the TME by TIC-secreted CXCL12 (57).MDSCs, TAMs, and Tregs are the three most prevalent immunosuppressive cells in the TME. In their symbiotic connections with TICs, they interact and suppress tumor immunity. In the next sections, we shall discuss these cells’ interactions with TICs in greater detail. This article outlines the possible paths of TIC function, their upstream transcription factors, and the interaction between TICs and immunosuppressive cells in **Figure 1**.

#### TICs and MDSCs

MDSCs are one of the most discussed biological entities in immunology. Despite differences in their classification and history, MDSCs are most commonly used to describe cells produced during chronic inflammation, particularly in late malignancy, and they contain T-cell immunosuppressive capabilities (58). MDSCs are composed of a heterogeneous population of myeloid cells, among which granulocytic or polymorphonuclear cells (PMN-MDSCs) and mononuclear cells (M-MDSCs) constitute subpopulations (59). PMN-MDSCs comprise over 80% of all MDSCs, whereas M-MDSCs can differentiate into TAMs (60). MDSCs are the primary organizers of inflammation associated with cancer because of their dynamic expression of several polarized inflammatory programs that promote tumor growth, including tumor angiogenesis, immunosuppression, tissue remodeling, and the maintenance of TIC stemness (61). The development of cancer is aided by MDSCs’ promotion of angiogenesis, invasion, metastasis, and cancer cell survival (58, 62). According to research, MDSCs regulate the phenotypic transition in TICs. By upregulating the expression of microRNAs (miRNAs), which are short (~22 nt) non-coding endogenous RNAs that post-transcriptionally influence gene expression in cancer cells (63), MDSCs contribute to the TIC phenotype (64). In particular, MDSCs promote TIC-associated gene expression, spheroid formation, and cancer spread while suppressing T-cell activation. MDSCs cause cancer cells to express miRNA-101. The corepressor gene C-terminal binding protein-2 (CtBP2), which specifically targets stemness core genes, is then suppressed by miRNA-101. higher tumorigenicity, higher metastatic potential, and increased stemness of cancer cells are the results of this suppression. Interestingly, poor survival is independently predicted by both increased tumor miRNA-101 expression and greater MDSC infiltration. These components function independently of one another, and a lower prognosis is also associated with decreased CtBP2 expression in malignancies (64). MDSCs can stimulate mesenchymal development and TIC proliferation (65). The method through which MDSCs promote TIC stemness depends on STAT3. Reprogramming brought on by STAT3 phosphorylation can give monocytes a pro-tumor immunosuppressive phenotype. By blocking STAT3, MDSC’s ability to produce TICs *in vitro* can be completely eradicated (65). Additionally, MDSCs can promote stemness in TICs and upregulate PD-L1 by activating the PI3K/AKT/mTOR pathway (66). On the other hand, TICs draw in and encourage MDSC infiltration, development, and activation via secreting soluble chemicals and exosomes specific to different cancer types. These consist of granulocyte-macrophage colony-stimulating factor (GM-CSF), TGF-β, IL-8, and macrophage migration inhibitory factor (MIF) (67–69). Additionally, interaction between MDSCs and macrophages has been shown in other investigations to polarize macrophages toward an M2 phenotype (70).

#### TICs and TAMs

TAMs are incredibly flexible cells that undergo many forms of functional activation in response to a range of stimuli. Infiltrating macrophages, whose activity is influenced by inflammatory and stress signals within the TME, can mediate tumor immune evasion (71). TAMs suppress anti-tumor immune responses by preventing CD8+ T lymphocytes from entering tumor sites or by decreasing their cytotoxic activity (72). The two primary populations of TAMs are M1 macrophages, which repress the tumor, and M2 macrophages, which promote growth (73). By secreting CXCL8, which in turn activates NF-κB and PI3K/AKT signaling, TICs may maintain their survival, proliferation, and self-renewal through cell-intrinsic pathways. Simultaneously, CXCL8 induces activation in TAMs via the CXCR2-JAK2/STAT3 axis, promoting an M2-like TAM phenotype through paracrine, cell-extrinsic pathways (74). TICs in liver cancer generate IL-34, a gene that p53 transcriptionally suppresses, as a result of p53 depletion. By increasing CD36-mediated fatty acid oxidation metabolism, IL-34 promotes M2-like polarization of TAMs. These IL-34-coordinated TAMs promote immunological evasion by inhibiting CD8+T cell-mediated anti-tumor immunity (75). Using soluble mediators including IL-6, TGF-β, WNT ligands, and pleiotrophic trophic proteins, or juxtacrine signaling discovered in co-culture experiments, TAMs can, on the one hand, cause phenotypic alterations and maintain stemness in TICs (76–80). Through the activation of nuclear factor-κB (NF-κB), AKT, and signal transducer and activator of transcription 3 (STAT3), TAMs promote self-renewal by signaling to TICs via ephrin type-A receptor 4 (EPHA4) and receptor-type tyrosine-protein phosphatase ζ (PTPRZ1) (78, 79). Pancreatic tumors respond better to chemotherapy and have fewer TICs when TAMs are targeted (81). In conclusion, factors, exosomes, or metabolites produced by TICs attract and polarize TAMs (82, 83). However, the promotion of TICs stemness and phenotypic alterations depends on TAM-derived paracrine substances or the physical interactions between TAMs and TICs (84, 85).

#### TICs and T cell

IL-17, which is released by T-helper 17 (Th17) cells, promotes TIC self-renewal and stemness maintenance. Both IL-17 stimulation and IL-17 introduction significantly enhance the growth of cancer and the ability to form spheroid in a dose-dependent manner. Furthermore, nude mice with higher levels of IL-17 gene expression are more carcinogenic (86). An important component of the TME, regulatory T cells (Tregs) can enhance carcinogenesis, metastasis, and chemotherapeutic drug resistance in cancer cells, as well as stimulate tumor angiogenesis and reduce anti-tumor immunity (87). There may be a connection between Tregs and TICs since, whereas TICs can draw Tregs into the TME, regs also support TIC stemness (87). Higher Th1 CD4+ T cell and/or CTL densities are associated with longer overall survival (88). These T-cell populations produce high quantities of the proinflammatory cytokine interferon-gamma (IFNγ), which is crucial for both local and systemic immunity (89). With its cytotoxic impact, immunostimulatory properties, and cell proliferation inhibitory effects, IFNγ is one of the primary mediators of anti-tumor immunity (90). However, studies have demonstrated that T-cell-derived IFNγ induces TICs to display certain functional traits, including as spheroid formation and resistance to chemotherapy or radiation, in addition to increasing stem cell markers (91). On the other hand, by upregulating programmed death-1 (PD-1) on CD8+ T-cells, TIC-produced PD-L1 can encourage CD8+ T-cell exhaustion (92).

### Checkpoint molecule expression

TIC-secreted exosomes trigger the STAT3 pathway, which raises PD-L1 in TAMs (83). MDSCs can promote the stemness of TICs and raise PD-L1 by activating the PI3K/AKT/mTOR pathway (66).By stimulating the ITGB4/SNAI1/SIRT3 signaling pathway, PD-L1 can encourage tumor growth and metastasis, suggesting yet another potential mechanism for these pathways to work in concert (93). Prior studies have shown that the TIC-like population of malignancies, including colorectal cancer, has higher levels of PD-L1 (94). Through a STT3-dependent mechanism, PD-L1 preferentially expresses on TICs and aids in their immune evasion (95). This finding suggests a possible way that TICs evade immune surveillance. These results demonstrate the therapeutic potential of employing immune checkpoint inhibitors to target TICs. One intriguing approach to creating novel therapies that cause cancer patients’ tumors to partially or completely recede is immune checkpoint inhibition (96). Research suggests that checkpoint blockade therapy is highly effective in treating immunogenic malignancies with high levels of CTL infiltration (97). In particular, T-cell activation and proliferation potential can be restored by anti-PD-1 or anti-PD-L1 antibodies, which will allow immune cells to detect and eradicate cancerous cells more efficiently (98). Patients with PD-L1-positive tumors have much greater response rates to PD-1/PD-L1 blocking therapy than patients without PD-L1 expression, according to clinical evidence (99). Importantly, preclinical studies demonstrate that PD-1/PD-L1 blockade synergistically enhances the antitumor efficacy of TIC-targeted vaccines in murine cancer models (100).

### Low immunogenicity

TICs surviving persistent immune pressure must circumvent genomic alterations that might induce novel innate and adaptive immune responses, ultimately manifesting an immunogenically attenuated phenotype (101). Comprehensive characterization of TIC-associated immune profiles represents a fundamental prerequisite for developing successful TIC-targeted immunotherapies. These immunological attributes - encompassing antigen processing/presentation machinery (including MHC complexes encoded by HLA genes), costimulatory/inhibitory signaling molecules, tumor-associated antigens (TAAs), and cytokine networks - represent critical determinants for effective immunotherapy development. The diminished MHC I expression observed in TICs may promote their survival through impaired T-cell recognition (102). TICs displaying attenuated MHC I presentation or deficient natural killer cell-activating ligand expression demonstrate impaired immune recognition, potentially conferring dual resistance to chemotherapy and immune-mediated elimination (103). Moreover, TICs exhibit systemic downregulation of antigen processing components including transporter associated with antigen processing (TAP), low molecular weight proteasome subunits (LMP), and β2-microglobulin (104). Immune profiling studies have identified distinct expression patterns of costimulatory (e.g., CD80/CD86) and coinhibitory (e.g., CTLA-4, PD-1/PD-L1, B7-H2/H3) molecules in TICs, revealing a predominant inhibitory signature with costimulatory molecule deficiency (105). This immunological tolerance is compounded by the weak immunogenicity of TAAs derived from TICs, which exhibit heterogeneous expression patterns within tumor masses due to immune selection pressures (106). Consequently, identifying novel tumor-specific neoantigens that stably associate with malignant progression and evade host immune editing remains a central challenge in immunotherapy development. Notably, soluble mediators in the TIC secretome warrant particular attention. Cytokines including CCL-2, IL-6, IL-8, IL-10, IL-13, and TNF-α show tumor-specific secretion profiles, with experimental evidence demonstrating TIC-derived cytokines mediate immune evasion through recruitment and activation of MDSCs and TAMs (107).

## Strategies to target TICs

### Inhibition of TIC-specific pathways

Numerous pluripotency-associated transcription factors, such as OCT4, SOX2, NANOG, KLF4, and MYC, regulate TICs. Experimental evidence indicates that TIC-derived cytokines mediate immune evasion through recruitment and activation of MDSCs and TAMs. Cytokines, such as CCL-2, IL-6, IL-8, IL-10, IL-13, and TNF-α, exhibit tumor-specific secretion profiles (108). Important signaling pathways, including Notch, Hedgehog (Hh), Wnt/β-catenin, FGF/FGFR, EGF/EGFR, NF-κB, MAPK, PTEN/PI3K, HER2, JAK/STAT, PI3K/AKT/mTOR, TGF/SMAD, and PPAR pathways, are also commonly dysregulated in TICs (108–111). Activation of the STAT3 and Notch3/mTOR pathways promotes higher PD-L1 expression in TICs than in non-TICs, especially in colorectal cancer, gastric cancer, and other gastrointestinal cancers. While STAT3 suppression has been demonstrated to restore T-cell function (112), PD-L1 overexpression increases TIC stemness through a self-sustaining positive feedback loop (113, 114). By successfully removing lung cancer TICs, lowering metastasis, and extending patient survival, napabacusin, a STAT3 inhibitor, has shown strong efficacy in clinical trials (115). One important mediator of TIC-TME interactions is NF-κB, whose suppression significantly lowers the expression of cancer stem cell markers (116). The transcription factor SOX2 orchestrates transcriptional networks that sustain cellular stemness (117) and confer anti-apoptotic capabilities (118). and it plays essential roles in maintaining both embryonic stem cell features and TIC characteristics (119–121). Numerous cancer types have been shown to have SOX2 as an oncogenic driver that is enhanced during carcinogenesis, metastasis, and recurrence (122, 123). *In vivo*, tumor growth and chemoresistance are greatly reduced when SOX2 is suppressed (123). By working in tandem with SOX2, the Wnt/β-catenin pathway plays a vital role in maintaining cancer stemness. SOX2 improves β-catenin’s nuclear localization and transcriptional activity by directly interacting with it (123, 124). An almost universal characteristic of sporadic colorectal cancers is aberrant Wnt/β-catenin signaling, which is mostly caused by APC mutations. Nuclear β-catenin levels rise as a result of pathway activation, which promotes the development of the T-cell factor/lymphoid enhancer factor complex and the subsequent upregulation of target genes such as AXIN2, SOX2, TCF7, c-MYC, and MMP7 (125, 126). By inducing TIC apoptosis, therapeutic blocking of this mechanism significantly lowers tumor recurrence (127–129). Numerous cancers have been shown to have deregulation of JAK/STAT signaling in TICs (130–132). By inhibiting STAT1 activation, clinical intervention with FDA-approved JAK1/2 inhibitor ruxolitinib or anti-IFNγ antibodies successfully stops IFNγ-mediated TIC production (91). Together, these results highlight the therapeutic potential of focusing on TICs’ aberrant signaling pathways to enhance clinical results.

### Differentiation therapy

TICs exhibit characteristic stem cell characteristics, such as the ability to self-renew and the potential for multi-lineage differentiation, as a unique subpopulation within tumor heterogeneity. These cells promote the development of primary tumors, mediate resistance to treatment, and aid in distant metastases and tumor recurrence (133). According to new data, TICs are malleable cells that can switch between quiescent and proliferative states in both directions (134). By using pharmaceutical intervention to reroute tumor reprogramming toward terminal differentiation or death while reducing proliferative potential, differentiation treatment takes advantage of the reversible differentiation abnormalities seen in malignant cells (135, 136). The goal of the TIC differentiation therapeutic paradigm is to either cause TICs to mature terminally or transform them into therapy-sensitive non-mesenchymal equivalents. This approach seeks to reduce invasive potential and malignant development by converting aggressive, undifferentiated tumors into differentiated cell populations with reduced tumorigenicity. In the end, these differentiation-based strategies might eliminate tumors and exhaust the TIC reservoir (137). Genetically defined models provide mechanistic insights, but translational hurdles in solid tumors continue because of sample heterogeneity and constraints in *in vitro* culture. Targeted inhibition of this oncogenic chimeric protein with functional antibodies causes TIC differentiation and functional ablation in PTPRK-RSPO3 fusion-driven colon cancers, which is a noteworthy example (138).This proof-of-concept illustrates how differentiation treatment may be used to treat specific solid gastrointestinal cancers with distinct driver changes.

### Enhancing immune recognition

TIC-specific antigens or surface markers, including as CD44, CD133, and EpCAM, can be targeted by vaccines and adoptive cell treatments (such CAR-T cells). Using embryonic stem cells as universal preventive cancer vaccines is a unique treatment approach made possible by the antigenic similarities between TICs and these cells. Anti-embryonic antigens can induce anti-tumor immune responses through cross-reactive immunity (139), as prior studies have confirmed that tumor-embryonic antigens (such as carcinoembryonic antigen) are co-expressed in both TICs and embryonic stem cells (140). In addition to being a rich source of TIC-specific antigens, embryonic stem cells have the ability to imitate embryonic niche conditions in their conditioned medium, which can aid in differentiation-based cancer therapy. As preventive measures against lung cancer (141),CRC (142) and ovarian cancer (143), inactivated embryonic stem cell-derived vaccines have been created with success and have shown effectiveness in inhibiting the growth and spread of tumors in animal models. In addition to inducing strong TIC-specific CTL responses, therapeutic cancer vaccines that target TICs have shown strong antitumor activity against SCC7 squamous cell carcinoma and D5 melanoma in mouse models (144). Targeting self-renewing TICs is one area in which chimeric antigen receptor (CAR)-T cell therapy, a novel immunotherapy strategy that involves genetically modifying T cells to express cancer-specific CARs (145), shows promise (146). In both syngeneic and xenograft murine models, preclinical research has shown that CAR-T cell therapy can completely eradicate established solid tumors and metastatic lesions in a variety of cancer types, including colon, breast, melanoma, and pancreatic cancer (147). Prior research has shown that Claudin18.2-targeted CAR-T cell therapy is remarkably effective in treating gastroesophageal junction cancer. With a median overall survival of 8.8 months and a disease control rate of 91.8%, the results of a phase II clinical trial (NCT04581473) revealed that some patients even experienced long-term remission (148). With a 6-month overall survival rate of 81.2%, Claudin18.2-targeted CAR-T cell treatment achieved an objective response rate of 57.1% and a disease control rate of 75.0% in patients with gastric cancer. According to these early findings, CAR-T cell treatment shows encouraging efficacy and tolerable safety in cancer patients (149).

### Overcoming immunosuppression

Even though tumors have an immunosuppressive environment where cancer cells can inhibit the activation of immune cells through a variety of mechanisms, including the recruitment of TAMs, MDSCs, and Tregs, the attenuation of MHC class I expression, and the use of the PD-1/PD-L1 axis, the immune response against TICs can be strengthened by modulating the TME by using inhibitors of immune checkpoint molecules or reducing the recruitment of immunosuppressive cells. Many drugs that target immune checkpoint receptors, such as CTLA-4, PD-1 (cemiplimab (150)) and PD-L1(avelumab (151), durvalumab (152)). The use of the antibody ipilimumab, which targets cytotoxic T-lymphocyte-associated antigen 4 (CTLA-4), was authorized in 2011. Additionally, tremelimumab (CP-675206), a fully human monoclonal antibody, binds to the CTLA-4 molecule expressed on the surface of activated T lymphocytes and T regulatory cells. Tremelimumab prevents CTLA4 from binding to its target ligands (B7–1 and B7-2) by inhibiting the negative regulatory signal that CTLA4 sends on T cell priming (153). 2014 also saw the approval of pembrolizumab and nivolumab, two antibodies that block programmed cell death protein 1 (anti-PD-1) and its ligand 1 (anti-PD-L1) (154). Blocking CTLA4, PD-1, and PD-L1 inhibitory effects can promote and enable effective immune responses against tumor cells. Tregs, a vital part of the immune system, are important targets for treatment and can be used to predict the course and prognosis of cancer (155). Tregs have been shown to directly kill NK cells via β-galactoside-binding protein, which promotes lung metastasis (156). To stop cancer cells from spreading to the lungs, it is enough to target Treg cells (157). MDSCs, recognized as one of the main cellular components in the tumor microenvironment, promote tumor growth by carrying out immunosuppressive functions. They are now significant modulators of the cancer immune response and targets for cancer therapy (158). Modifying the TAM response may enhance immunotherapy. Many strategies to reduce TAMs have been studied in laboratory settings and are thought to be effective therapeutic interventions at this time (159). By inhibiting M-MDSC and TAM survival, some anti-CSF1R drugs that are being studied in cancer patients have demonstrated promising anticancer potential (160). Immunoevasion linked to TICs may be caused by a variety of extrinsic stimuli in addition to immunosuppressive cell activity. Because of their resistance to degradation, persistent organic pollutants like perfluorooctanoic acid (PFOA) and perfluorooctanesulfonic acid (PFOS), which are widely used in industrial applications like firefighting foams and water-reactive materials, have become common environmental contaminants, raising concerns about their possible health effects. Per- and polyfluoroalkyl substances (PFAS) may facilitate immune evasion by altering tumor-associated gene expression, controlling immune cell function, and improving TIC properties, according to earlier research using network toxicology, single-cell sequencing, spatial transcriptomics, and molecular simulation technologies (161, 162).

## Combination therapies

### Checkpoint inhibitors + TIC targeting

Checkpoint inhibitor monotherapy has demonstrated the most significant activity in tumor types with high PD-L1 expression and/or high microsatellite instability or mismatch repair deficiency, though this is limited to no more than one-third of cancer patients authorized for checkpoint inhibitor treatment (163). Because CTLA-4 and PD-1 antagonists are more effective when used jointly than when used alone, a combined immunotherapy regimen was recently approved (164). While PD-1 inhibitors target peripheral T cell activation, especially in the tumor context, CTLA-4 antagonists affect T cell priming (165). The robust immune responses shown in *in vivo* studies when both targets are blocked have strengthened the theoretical foundation for combined ICB (166). In conclusion, if CD8+ T cells are absent from the tumor microenvironment, blocking the PD-1/PD-L1 pathway will not work. Combining this tactic with CTLA-4 blockage may increase the quantity of activated CD8+ T cells (167). However, combining two ICBs will unavoidably cause more side effects, and their clinical application might be challenging (168). For tumors with low immunogenicity or stromal fibrosis, where the effect of checkpoint inhibition as a monotherapy is minimal or nonexistent, combining checkpoint inhibition with other treatments may result in a synergistic response (169). Some researchers suggest combining TIC targeting with immune checkpoint blocking to improve treatment outcomes. As was previously known, TICs are commonly accompanied by aberrant route changes. By combining ICB with targeting these pathways, treatment resistance can be reduced and patient outcomes can be greatly enhanced. Using methods like organoids, transcriptomics, genomic sequencing, and immunohistochemistry, suitable individuals should be identified and monitored both before and during treatment (170). Research has demonstrated that blocking oncogenic Myc signaling using epigenetic strategies (like JQ1) significantly reduces PD-L1 expression, which is why anti-PD-1 antibodies and JQ1 produce a synergistic immune response (171). These results suggest that combining immune checkpoint blockade with TIC targeting can significantly improve the efficacy of immunotherapy for malignancies.

### Chemotherapy + immunotherapy/targeted therapy

Given the immunomodulatory and adjuvant effects of classical chemotherapy and its widespread clinical usage, combining immunotherapy with chemotherapy presents an enticing option to boost immunotherapy’s efficacy across a larger patient population (172). In particular, TIC-targeted chemotherapy seeks to either use surface receptors to transport chemotherapeutic drugs directly into TICs for efficient removal or disrupt basic intracellular pathways in TICs. The immunosuppressive TME is essential for preserving TICs’ stem-like characteristics. As a result, some immunotherapies that can rewire the TME have also shown promise in eliminating TICs (173). The use of combined treatment techniques is encouraged by the possibility for chemotherapy and immunotherapy medications to complement each other mechanistically, even though only a small number of patients benefit from either medication alone. Combining immunomodulators, like immune checkpoint inhibitors, can improve the antitumor immune response triggered by chemotherapeutic drugs while suppressing or even eliminating tumor growth and metastasis. Consequently, immunotherapy in combination with chemotherapy has recently been identified as a highly promising approach to improve these medications’ efficacy (172, 174). Pancreatic cancer (175), gastric cancer (176), CRC (177), and hepatocellular carcinoma (178) are among the gastrointestinal malignancies for which standard chemotherapy and immunotherapy have been shown to have beneficial therapeutic effects (179, 180). Intracellular proteins that are essential for the growth, development, or metabolism of TICs can be inhibited by chemotherapy drugs. For example, it has been demonstrated that ALDH1 inhibitors cause apoptosis in TICs and block the production of markers linked to stemness (181). Additionally, CD44-positive breast cancer TICs can be precisely targeted by gemcitabine-conjugated nanoparticles functionalized with CD44 antibodies, improving overall therapeutic efficacy (182). Glioblastoma TICs have been successfully eradicated by NK cell therapy in the field of immunotherapy (183). Furthermore, surface receptors on TICs have been used for targeted delivery of immunomodulators, much like chemotherapeutic medications and radioisotopes (184). There are intrinsic disadvantages to immunotherapy and chemotherapy, despite their notable clinical benefits. Chemotherapy drugs have the ability to directly destroy or inactivate dendritic cells and other immune cells (185). High-dose chemotherapy often results in a decrease in B cells, T cells, and NK cells (186). These cells possess every functional characteristic needed to expose effector T cells to antigens linked to tumors, hence stopping the formation of tumors in the host (187). The immunosuppression caused by the death of these cells may be a significant factor in decreased efficacy and tumor recurrence when immunotherapy and chemotherapy are used in combination.

### Clinical trials

Clinical studies of medications that target surface indicators linked to CSCs Monoclonal antibodies (mAbs) that target surface indicators unique to CSCs have become a cutting-edge cancer treatment tool. Future and ongoing clinical trials that combine immunotherapy with TIC-targeted medicines for malignancies of the digestive system will yield vital information to improve these strategies. Targeted treatments have shown considerable promise in treating TICs, particularly solid tumors, as seen by recent developments in clinical research. To overcome tumor heterogeneity, improve safety even more, and investigate earlier clinical applications, more research is required. Targeted treatments for TICs have advanced from proof-of-concept to clinical translation, notwithstanding the lingering obstacles. In order to get from “prolonged survival” to “cure of recurrence,” future research must optimize current therapies through interdisciplinary collaboration (immunology, AI, materials science) and investigate early interventions. In **Table 2**, we provide an overview of some of the current clinical research on GI cancers.

**Table 2:** TICs-directed immunotherapy in ongoing clinical trials.

Disease	Trial description	Enrollment (Estimated)	Phase	NCT Number	Current status
Gastric cancer	CAR-T	24	I	NCT06010862	Recruiting
	EpCAM- CAR-T	19	II	NCT02725125	Unknown status
	CAR-T	60	I	NCT05396300	Active, not recruiting
	IM92 CAR-T	6	Early I	NCT05275062	Unknown status
	CAR-T	60	I/II	NCT06006390	Recruiting
	CAR-T	50	I	NCT06821048	Recruiting
	CAR-T	60	I	NCT06126406	Recruiting
	EpCAM- CAR-T	60	I/II	NCT03013712	Unknown status
	CDH17 CAR T-cell	135	I/II	NCT06055439	Recruiting
	EpCAM- CAR-T	30	I	NCT02915445	Active, not recruiting
	CAR-T	24	Not Applicable	NCT03159819	Unknown status
Colorectal cancer	αPD1-MSLN-CAR-T	10	Early I	NCT04503980	Unknown status
	IM96 CAR-T	9	I	NCT06718738	Recruiting
	CAR-T	30	Early I	NCT06675513	not yet recruiting
	NKG2D CAR-T	9	Early I	NCT05248048	Unknown status
	CAR-T	18	Early I	NCT04513431	Unknown status
	CAR-T	40	Not Applicable	NCT05401318	Recruiting
	GCC19 CAR-T	30	I	NCT05319314	Recruiting
Pancreatic cancer	CAR-T	27	I	NCT06158139	Recruiting
	CAR-T	10	Early I	NCT03267173	Unknown status
	EX02 CAR-T	6	Early I	NCT06196658	not yet recruiting
	U87 CAR-T	12	I	NCT05605197	Recruiting
	CD276 CAR-T	10	I/II	NCT05143151	Unknown status
	CAR-T	80	Not Applicable	NCT04203459	Unknown status
	CAR-T	18	I	NCT03323944	Recruiting
Hepatocellular carcinoma	CAR-T	20	I	NCT04121273	Unknown status
	GPC3 CAR-T	15	I	NCT06461624	Recruiting
	GPC3-CAR T	30	I/II	NCT06641453	not yet recruiting
	CD19 CAR-T	12	I	NCT06676982	not yet recruiting
	CAR-T	50	Early I	NCT03672305	Unknown status
	B7H3 CAR-T	15	I/II	NCT05323201	Recruiting
	GPC3 CAR-T	38	I	NCT05003895	Recruiting
	GPC3 CAR-T	60	Early I	NCT06653023	Recruiting
	GPC3 CAR-T	12	Not Applicable	NCT05926726	Recruiting
	GPC3 CAR-T	30	I/II	NCT02715362	Unknown status
	CAR-T	3	I	NCT05131763	Unknown status
	GPC3 CAR-T	20	Not Applicable	NCT05620706	Recruiting

## Challenges and future directions

Many patients continue to get ineffective therapy despite advancements in cancer treatment, which results in disease progression, recurrence, and a worse overall survival rate. Limitations in basic research and obstacles in clinical trials are the two primary categories of current challenges. While clinical trials challenges primarily include unclear therapeutic targets, low clinical trial success rates, and a lack of specific biomarkers, basic research challenges primarily include a lack of knowledge about TIC heterogeneity, immune evasion mechanisms, and the limitations of current models.

One of the most crucial things to keep in mind is tumor heterogeneity. The identification of several clones with different DNA sequences within the same tumor has led to the widespread recognition of cancer as a heterogeneous illness (188). Intra-tumoral heterogeneity is increasingly understood to play a role in treatment failure and the advancement of disease (189). Such heterogeneity is regarded as a significant barrier to precision cancer therapy (190)and also results in reduced immune responses against cancer (191). Both genetic determinants (primarily involving developmental pathways through gene mutations and tumor microenvironment alterations) and non-genetic factors, such as epigenetic modifications like DNA methylation, histone modifications, chromatin accessibility, microRNAs, and other non-coding RNAs, are strongly implicated in functional heterogeneity (192–194). Furthermore, TIC-mediated immune evasion continues to be a major contributing factor to immunotherapy failure. TICs may generate more immune escape mechanisms as they mature. To comprehend these mechanisms and learn how to combat them, more research is required. Finally, few molecular targets have been effectively converted into clinical care, and clinical trials continue to have high failure rates, despite a great deal of attention being paid to identifying key molecular targets as possible avenues for therapeutic intervention against TIC activities.

Reliable biomarkers are still lacking in clinical research. Despite the development of certain TIC-associated indicators, their specificity is typically lacking. It is obvious that in order to target this cell population efficiently, a thorough understanding of TIC indicators is necessary. Further research is still required to assess and validate TIC markers. Furthermore, it might be necessary to create treatments that can eradicate TICs with a variety of genetic alterations and phenotypic traits (195).Immunotherapy failure may be caused by unclear treatment targets, and clinical translation is hampered by poor clinical trial success rates taken together.

In order to improve our knowledge of TIC-associated immune microenvironments and particular indicators to direct future study, future studies should concentrate on using single-cell and spatial transcriptomic technologies to map the immune microenvironment of TICs (196, 197). To overcome drug resistance and increase the effectiveness of immunotherapy for tumors of the digestive system like colorectal cancer, Hong et al., for example, proposed an integrative genomics and single-cell analysis framework to identify immune-related and potential drug targets in TIC-enriched populations (198). In order to define liver cancer heterogeneity and create risk stratification models, Yang et al. used multi-region sequencing in conjunction with spatial transcriptomics, offering mechanistic insights into clinical difficulties driven by TICs (199).Yang’s group recently created a computational pipeline that combines five statistical inference techniques and is named SiLi (Statistical Inference-based Synthetic Lethality Identification). Through SiLi analysis of large-scale sequencing datasets, they methodically discovered synthetic lethal interactions in liver cancer, providing possible approaches for creating analogous algorithms to create TIC-targeted synthetic lethal combination treatments that address metastasis and recurrence. Last but not least, researchers can trace the origins of tumor-initiating cells and pinpoint important regulatory pathways to guide future targeted therapies by fusing lineage tracing with CRISPR gene editing technology (200). Integrating multi-omics data to forecast treatment responses or immune evasion patterns across several tumors from intricate databases has become possible with the development of machine learning (201).

## Conclusion

Targeting tumor-initiating cells in tumors of the digestive system is a promising way to improve cancer immunotherapy. By addressing the challenges posed by TICs, such as immune evasion and treatment resistance, therapies can improve patient outcomes and reduce tumor recurrence. Combination techniques, especially those that combine immune control and direct TIC targeting, are likely to determine the future of cancer treatment.
